# Screening for central precocious puberty by single basal Luteinizing Hormone levels

**DOI:** 10.1007/s12020-024-03781-9

**Published:** 2024-03-20

**Authors:** Alessandra Li Pomi, Perla Scalini, Salvatore De Masi, Domenico Corica, Giorgia Pepe, Malgorzata Wasniewska, Stefano Stagi

**Affiliations:** 1 Department of Human Pathology of Adulthood and Childhood, University of Messina, Messina, Italy; 2 Meyer Children’s Hospital IRCCS, Florence, Italy; 3 Clinical Trial Center, Careggi University Hospital, Florence, Italy; 4 Pediatric Unit “G. Martino” University Hospital, Messina, Italy; 5 Health Sciences Department, University of Florence, Florence, Italy

**Keywords:** Precocious puberty, Puberty, Luteinizing hormone, LH, GnRH test, Diagnosis

## Abstract

**Purpose:**

To identify cut-off for basal LH levels and for pelvic ultrasound uterine and ovarian parameters indicating an Hypotalamic–Pituitary–Gonadal (HPG) axis activation as diagnostic of Central Precocious Puberty (CPP).

**Methods:**

248 girls referred for suspected precocious/early puberty who had undergone a GnRH stimulation test were enrolled and divided into three groups: Premature Idiopathic Thelarche (PIT), CPP, and Early Puberty (EA). For every patient basal serum Luteinising Hormone (LH) and Follicle Stimulating Hormone (FSH), basal LH/FSH ratio and pelvic ultrasonographic parameters were also collected. Through the use of Receiver Operating Curves (ROCs) the sensitivity (Se) and specificity (Sp) of basal LH, FSH, LH/FSH ratio and ultrasonographic parameters were evaluated at each level and Area Under the Curve (AUC) was measured.

**Results:**

Basal LH model ≥0.14 mIU/mL reached the highest predictability (90.6% and 78.2%, Se and Sp, respectively). Basal LH/FSH ratio ≥0.1 showed a sensitivity of 85.90% and a specificity of 78.14%, while basal FSH cut-off (≥2.36 mIU/mL) had the lowest predictability, with a less favourable sensitivity (71%) and specificity (70.5%). Cut-off point for uterine length as 35 mm, (83.5% and 42.9% of Se and Sp, respectively) was calculated. For ovarian volumes, ROC curves showed very low sensitivity and specificity.

**Conclusion:**

A single basal LH measurement under the cut-off limit may be adequate to exclude an HPG axis activation as CPP.

## Introduction

Puberty is a complex phenomenon occurring over several years with concomitant physical, hormonal, metabolic, psychological, and behavioural changes [1]. Physical pubertal changes are mainly the appearance of secondary sexual characteristics, the growth of external and internal genitalia, and changes in growth patterns [1]. In girls normal puberty onset is defined as the development of pubertal signs between 8 and 13 years [1].

During normal puberty, inhibitory mechanisms affecting the pulsatile Gonadotropin Releasing Hormone (GnRH) secretion decline, lead to increased pulsatile Luteinizing Hormone (LH) and Follicle-Stimulating Hormone (FHS) secretion [1]. Pubertal LH secretion is characterized by high levels, first at night and later during the day [1, 2]. This secretion leads to higher levels of sex hormones in pubertal than in pre-pubertal subjects, and eventually to the appearance of pubertal signs.

Clinically, Hypothalamic-Pituitary-Gonadal (HPG) axis activation in children can be documented by the finding of pubertal signs, which are defined by Tanner stages [3], associated with an accelerated growth rate and advanced bone maturation.

In girls, breast development is caused by oestrogen secreted by the ovaries under pituitary stimulation, while the growth of pubic hair is mainly due to the influence of adrenal androgens. The stage of breast development usually correlates well with the stage of pubic hair but since different endocrine organs control these two processes, the stages of each should be classified separately [4].

Precocious puberty is defined as development of breasts in girls under 8 years of age, whereas the appearance of first pubertal signs between 8 to 9 years defines Early Puberty (EP) [5]. Precocious puberty is usually caused by premature activation of the hypothalamic GnRH pulse generator (Central Precocious Puberty- CPP). This condition is associated with growth velocity acceleration and early epiphyseal maturation, potentially causing compromised final height [6, 7], as well as psychological stress [8]. For these reasons, early diagnosis and treatment are of paramount importance [7, 9].

CPP may resemble Premature Idiopathic Thelarche (PIT), which is also characterized by isolated early breast development under 8 years of age, but without growth acceleration or bone maturation, and which doesn’t require any treatment [10]. PIT is regarded as a normal variant of development and is not considered pathological [10]. The incidence of this condition is highest in the first year of life, with a second peak after the fifth year [11].

Differentiation between CPP and PIT is based on physical examination, bone age (BA) assessment, growth velocity and the GnRH stimulation test. It can be challenging to distinguish between early stages of CPP and PIT. The increased prevalence of obesity has made differential diagnosis even more difficult [12, 13].

The gold standard for detecting HPG axis activation is to measure the maximal LH level after a GnRH stimulation test [14–17]. Conventionally, a peak LH value of ≥5 mIU/mL is considered significant for activation of the hypothalamic GnRH pulse generator [16–18]. This test is mandatory both for diagnosing precocious or early puberty and for deciding if therapy with GnRH analogue is appropriate.

The GnRH stimulation test does, however, have some limitations. Despite its high specificity, its sensitivity is relatively low [19, 20], mainly due to a late transition to an LH-predominant response, typical of the puberty, in respect to the FSH-predominant response of the premature thelarche during the clinical progression of central precocious puberty [19]. There is disagreement about which criteria should be applied for its interpretation [21, 22]. The GnRH stimulation test is also costly and requires multiple blood sampling over long time periods.

Ultrasound (US) assessment of uterus and ovaries could be a useful tool for monitoring pubertal progression in girls [16, 17, 23–25]. Pelvic ultrasound is a non-invasive, rapid and reliable way of imaging the internal genitalia of girls and several studies have assessed the growth of the uterus and ovaries during childhood and adolescence [26]. A number of studies have attempted to evaluate the use of pelvic ultrasonography in differentiating between normal girls and girls with CPP [25, 27, 28], since uterine and ovarian volume seems to be stable until the beginning of pubertal development. A recent consensus statement confirmed that pelvic US imaging is helpful as an adjunct to the GnRH stimulation test in differentiating CPP and PIT. The statement reported cut off values for uterine length ranging from 34 to 40 mm and between 1 and 3 ml for ovarian volume [18]. However, although uterine and ovarian dimensions are significantly higher in girls with true CPP than in control subjects and girls with PIT, there is a significant overlap of normal pre-pubertal and early pubertal US parameters [23].

In this context many clinical and laboratory factors that could predict positive results on GnRH stimulation test have been studied in order to improve patient selection and timing for the test [29]. Particular attention has been given to basal levels of LH and FSH as markers of activation of the HPG axis in girls suspected to have central precocious puberty, but the results are controversial [21, 30–32]. The role of pelvic US in assessing the presence of CPP is debated [23–25, 27, 28].

The aim of this study is to identify cut-off limits for basal LH levels and for pelvic ultrasound uterine and ovarian parameters indicating HPG axis activation in order to decrease the need for GnRH stimulation tests.

## Materials and methods

Four hundred fifty-four patients (mean age 8.57 ± 2.30 years) were assessed at the Meyer Children’s University Hospital of Florence, Paediatric Endocrinology Unit and Outpatient clinic of Paediatric Endocrinology of University Hospital of Messina from October 2008 to March 2016 for pubertal disorders. From this population, we decided to study girls aged 4 to 9 years referred to our centre for the early appearance of breast development with Tanner stage 2 and 3. There were 248 girls (mean age 7.92 ± 0.83 years) in this group. In all patients, clinical examination at the baseline visit involved recording height, weight and body mass index (BMI) and pubertal staging. Endocrine evaluation included measurement of basal LH, FSH and LH/FSH ratio, and US examination of the uterus and ovaries. The patients also underwent a GnRH stimulation test to evaluate HPG axis activation. We defined HPG activation as a peak LH concentration of ≥5 mUI/mL on GnRH stimulation test [18].

Girls were divided into three groups according to age and GnRH test results. Girls in whom breast bud development occurred before 8 years of age with pre-pubertal height velocity and a pre-pubertal response to the GnRH test were diagnosed with PIT. Girls who developed breast buds before 8 years with accelerated height velocity and confirmed pubertal response to the GnRH test were diagnosed with CPP. Girls with breast development between 8 and 9 years and an LH peak ≥5 IU/L after a GnRH stimulation test were diagnosed as EP.

Patients diagnosed with peripheral puberty, such as those with McCune-Albright syndrome or non-classical congenital adrenal hyperplasia, or patients with a history of pelvic surgery, chemotherapy and/or pelvic radiotherapy, with early or precocious puberty caused by an intracranial lesion, chronic illness and/or long-term medication which might have affected the HPG axis (i.e., sexual steroids or GnRH-analogues) were excluded.

The study was conducted according to the Declaration of Helsinki and the European Guidelines on Good Clinical Practice. Ethical approval was obtained from the Regional Paediatrics Ethics Committee (approval number: 05/04/2016–48/2016). Written informed consent was obtained from parents and patients according to age and ability to consent.

### Auxological and clinical methods

Height was measured using a wall-mounted stadiometer, and weight was measured to the nearest 0.1 kg. All measurements were performed by the same trained staff members. The coefficient of variation (CV) values is <1% for these measurements. We calculated the BMI as the weight in kilograms divided by height in metres squared (kg/m^2^). Age-related reference values for height, weight and BMI were obtained from specific Italian growth charts [33]. Height and BMI were normalized for chronologic age by calculating standard deviation score (SDS), as previously reported [34]. SDS values were calculated according to the following formula: (patient value—mean of age-related reference value)/standard deviation of the age-related reference value [34]. Pubertal staging was performed according to Tanner and Whitehouse’s criteria [3].

The GnRH test was performed by taking basal serum samples of LH and FSH before injecting GnRH (T0) and then at the 15th, 30th, 45 h and 60th minutes following intravenous administration of 100 μg/m^2^ (maximum 100 μg) synthetic GnRH (Lutrelef 0.8 mg/10 mL, Ferring S,p.A., Italy) [35]. The peak LH and FSH were reported as the highest measurements of LH and FSH under GnRH stimulation. We considered activation of the hypothalamic GnRH pulse generator when the subject has a peak LH value of ≥5 mIU/mL on the GnRH test. According to that, we defined central precocious, early or normal puberty onset when there was a peak LH value of ≥5 mIU/mL on the GnRH test [18] and a ratio of stimulated LH/stimulated FSH of more than 1.0 [36]. However, it should be taken into account that each patient has had their own peak and each peak might have happened in a different moment, with no standardization (e.g., considering LH peak at 30 or 60 min).

### Laboratory methods

All laboratory measurements were performed on blood samples collected after overnight fasting. Plasma FSH and LH were measured by chemiluminescent immunometric assays using commercially available kits for the IMMULITE 2000 Systems analyser (Siemens Healthcare Diagnostics, Los Angeles, CA, USA). The lower limit of sensitivity for LH and FSH is 0.1 IU/L. The intra-assay coefficient varies from 2.6% to 8.5% whereas the inter-assay coefficient varies from 3.7% to 11.9%.

### Pelvic ultrasonography

Pelvic ultrasonography was performed at the time of the initial assessment using a Siemens Sonoline Elegra (Siemens, Issaquah, WA, USA) sonograph and a 6.5 MHz probe. Clear fluids were given to all the subjects so that all patients were scanned with a full bladder, which served as an acoustic window through which the pelvic organs were examined. When available, previous data about uterine length were collected. The volume (V) of the ovaries was also calculated by the ellipsoid formula, as follows: V (cm^3^) = longitudinal diameter (cm) x anteroposterior diameter (cm) x transverse diameter (cm) x 0.523. In pre-pubertal subjects, the normal ovarian surface volume is <2 ml [37], while uterine shape is tubular and longitudinal diameter is smaller than 35 mm.

### Statistical analysis

All data are expressed as means ± standard deviation (SD) or median at Q1 and Q3; *p* < 0.05 was considered to be statistically significant. The parametric sample analysis of variance was used for separate group comparisons with normal distribution, and group comparisons with non normal distribution were analysed using the non-parametric Kruskal–Wallis test. The sensitivity and specificity of gonadotropins and ultrasonographic parameters at each level were evaluated using Receiver-Operating Curves (ROCs), and the Area Under Curve (AUC) was measured. Youden’s J index [(sensitivity + specificity)-1] combined with clinical evaluation was then used to determine the cut-off points from the ROCs [38]. In a ROC the true positive rate sensitivity is plotted as a function of the false positive rate (100-specificity) for different cut-off points. Each point on the ROC represents a sensitivity/specificity pair corresponding to a particular decision threshold. A test with perfect discrimination (no overlap in the two distributions) has a ROC curve that passes through the upper left corner (100% sensitivity and 100% specificity). Therefore, the closer the ROC is to the upper left corner, the higher the overall accuracy of the test [39]. Statistical analysis was performed by STATA Statistical Software (STATA Corp., Vers.11).

## Results

Clinical features and hormone levels divided into three groups (CPP, EP, PIT) are presented in Table 1.

**Table 1:** Clinical features, basal LH, FSH and LH/FSH levels and uterine length and ovarian volumes measured by pelvic US according to final diagnosis

	CPP (*n* = 125)	EP (*n* = 45)	PIT (*n* = 78)	*p*	TOT
Chronological age, year	7.80 ± 0.65	8.70 ± 0.15	7.68 ± 1.03	<0.0001	7.92 ± 0.83
Height SDS	0.94 (0.14–1.67)	0.68 (0.08–1.43)	0.49 (−0.23–1.2)	<0.05	0.75 (0.03–1.41)
BMI SDS	0.22 (−0.14–0.74)	0.24 (−0.89–0.88)	0.44 (−0.02–1.07)	NS	0.42 (−0.07–1)
Uterine length, mm	39.39 ± 6.44	41.72 ± 8.64	35.5 ± 5.25	<0.0001	39.01 ± 6.97
Ovarian vol., right, mL	2.60 ± 1.42	2.57 ± 1.43	2.01 ± 1.54	<0.05	2.42 ± 1.48
Ovarian vol., left, mL	2.82 ± 1.72	2.42 ± 1.48	1.76 ± 1.18	<0.0001	2.42 ± 1.58
Basal LH, mIU/mL	0.52 (0.23–1.09)	0.46 (0.20–1.21)	0.10 (0.10–0.12)	<0.0001	0.5 (0.10–1.74)
Basal FSH, mIU/mL	3.38 (2.21–5.33)	3.20 (2.03–4.52)	2.02 (1.1–2.61)	<0.0001	2.66 (1.86–4.34)
Basal LH/FSH ratio	0.13 (0.09–0.21)	0.15 (0.10–0.32)	0.05 (0.02–0.08)	<0.0001	0.10 (0.05–0.21)

Data are expressed as mean ± standard deviation or medians (Q1–Q3), as indicated

*SDS* Standard Deviation Score, *LH* Luteinizing Hormone, *FSH* Follicle-Stimulating Hormone, *CPP* Central Precocious Puberty, *PIT* Premature Idiopathic Tealarche, *EP* Early Pyberty

Two hundred and forty-eight girls underwent a GnRH stimulation test for the early appearance of breast buds, among them 125 were diagnosed with CPP, 45 with EP and 78 with PIT. The mean age at the GnRH test was 7.92 ± 0.83 years.

Fifty-three patients with CPP, 7 patients with EP and 56 with PIT had Tanner stage 2, while 72 patients with CPP, 38 patients with EP and 22 patients with PIT had stage 3. At diagnosis, height SDS was significantly different among the groups, while BMI SDS was not (data not shown). Moreover, the uterine length and ovarian volumes were significantly increased in patients with CPP and EP compared with PIT (*p* < 0.0001 and < 0.05, respectively).

The basal levels of LH, FSH and basal LH/FSH ratio were all significantly higher in the CPP and EP groups compared with PIT. A certain overlap was observed among the groups for gonadotropin basal levels: 11 girls among the CPP group had LH basal serum levels within the basal LH serum levels of the PIT group and 36 CPP girls had FSH basal levels within the FSH basal levels of the PIT group, while there was no overlap between CPP and PIT basal LH/FSH range levels. The same tendency was noted in the EP group: 4, 15 and 1 girls had LH, FSH and LH/FSH basal serum levels within the PIT LH, FSH and LH/FSH basal serum range, respectively.

**Fig. 1:**
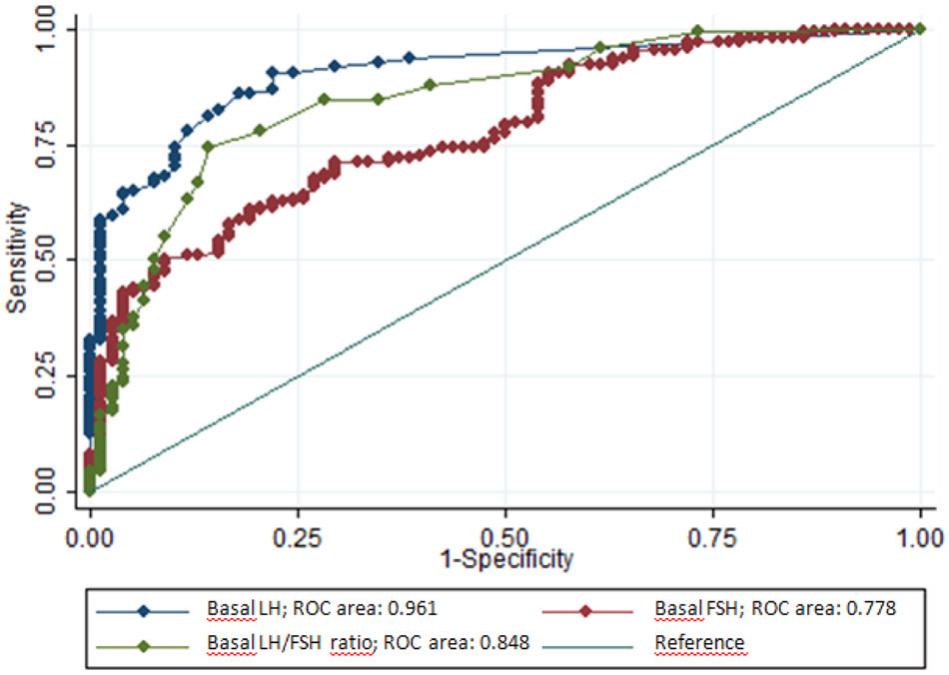
ROCs of basal LH levels, FSH levels and LH/FSH ratio for predicting HPG axis activation. LH Luteinizing Hormone, FSH Follicle-Stimulating Hormone

Considering LH after GnRH stimulation ≥5 mIU/mL as diagnostic for CPP, ROCs for basal LH, FSH and LH/FSH ratio were constructed and AUC was measured for each curve (Fig. 1).

The best cut off points based on sensitivity and specificity for diagnosing HPG axis activation was then determined for LH, FSH and LH/FSH ratio and were 0.14 mIU/mL, 2.36 mIU/mL, 0.1, respectively. The basal LH model, with an AUC of 0.906, reached the highest predictability (90.6% and 78.2%, sensitivity and specificity, respectively), while the basal FSH model had the lowest, with a less favourable sensitivity (71%) and specificity (70.5%). The sensitivities and specificities of these cut-off points are shown in Table 2.

**Table 2:** Sensitivity and specificity for basal LH, FSH and LH/FSH cut-off points discriminating HPG axis activation

Hormone	AUC (95% CI)	Cut-off points	Sensitivity	Specificity
LH (mIU/mL)	0.906 (0.87–0.94)	0.14	90.6%	78.2%
FSH (mIU/mL)	0.779 (0.72–0.84)	2.36	71.01%	70.51%
LH/FSH ratio	0.848 (0.79–0.90)	0.1	85.90%	78.14%

*LH* Luteinizing Hormone, *FSH* Follicle-Stimulating Hormone

It is important to note that 61 out of 78 girls within the PIT group (true negative 78.2%) demonstrated basal LH levels under the cut-off limit of 0.14 mIU/mL, while 11 out of 125 CPP girls and 5 out of 45 girls in the EP group had basal LH values < 0.14 mUI/mL (false negative 8.8% and 11.1%, respectively), probably due to the low LH production early in puberty [40].

**Fig. 2:**
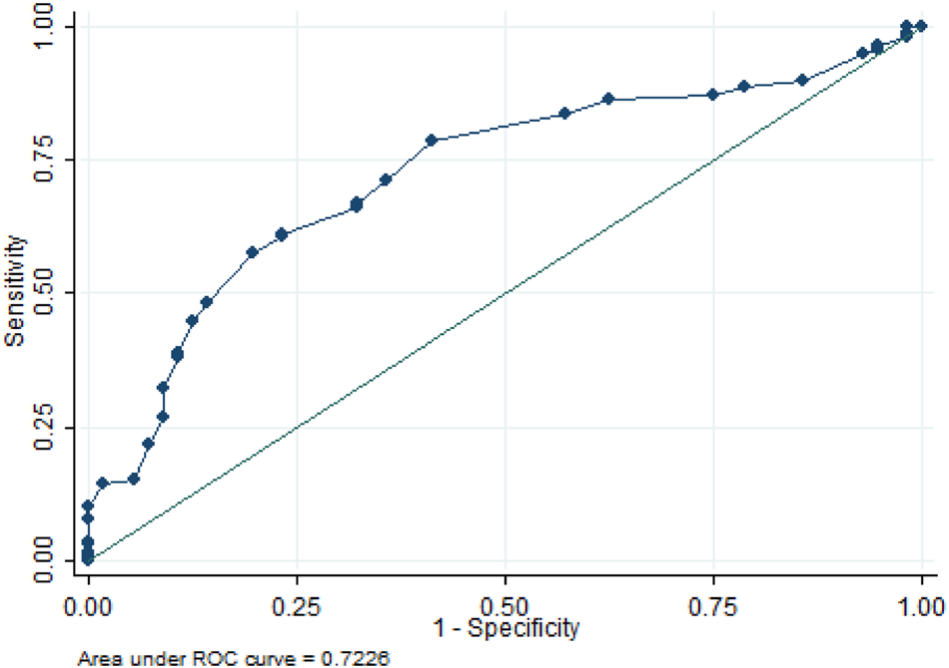
ROC curve for uterine length in detection of HPX axis activation

For US findings, we calculated cut-off point for uterine length (Fig. 2) as 35 mm, (83.5% and 42.9% of sensitivity and specificity, respectively). For ovarian volumes, ROCs analysis showed very low sensitivity and specificity, so we chose a volume of 2 ml as cut-off, according to the literature [39].

Based on the previous evaluations we defined a patient to have a positive pelvic ultrasonographic finding if at least one parameter, either uterine length or ovarian volumes, was above the cut-off limits. Sensitivity and specificity of an evaluation comprising both LH basal levels ≥0.14 mIU/mL and positive pelvic ultrasonographyc parameters were 82.1% and 83.1%, respectively. A test comprising both LH ≥ 0.14 mIU/mL and LH/FSH ratio ≥0.1 reached a sensitivity of 74.0% and a specificity of 93.6%.

## Discussion

The number of patients referred to paediatric endocrinology for signs of early pubertal is increasing.

Early diagnosis and treatment are critical for patients with CPP to avoid compromising adult height and psychological consequences [8, 16, 17, 41, 42], while PIT does not require immediate treatment but only follow up. Differential diagnosis depends on clinical evaluation, radiological studies (bone age assessment, pelvic ultrasonography) and laboratory assessment.

The GnRH stimulation test is the gold standard for diagnosing CPP since it has almost 100% specificity despite its low sensitivity [16, 17, 19, 20]. However, this test is expensive, time consuming and painful, since it requires an intra-venous access placement for several blood samples in a day hospital setting.

For these reasons, many authors over the years have investigated the possible role of gonadotropins basal levels in CPP diagnosis [21, 30, 31, 43–48]. Different LH basal cut-offs proposed in the literature are presented in Table 3.

**Table 3:** Basal LH cut-off levels proposed in available study

	Basal LH cut-off (mIU/mL)	Sensitivity (%)	Specificity (%)
Present study	0.14	90.6	78.2
Binay C et al. [40]	0.12	79	91
Lee DS et al. [27]	0.1	88.4	56.4
Pasternak Y et al. [28]	0.1	64	94
Houk CP et al. [39]	0.1	93	100
Neely EK et al. [18]	0.1	94	88
Chotipakornkul et al. [45]	0,1	76.8	90
Heo et al. [46]	0.245	88	48

*LH* Luteinizing Hormone

In our study, we identified 0.14 mIU/mL as the best cut-off point for basal LH, with a sensitivity of 90.6% and a specificity of 78.2%. This cut-off is similar to that proposed by Binay et al. of 0.12 mIU/ mL [44], although this study found a higher specificity (91%) and a lower sensitivity (79%) with a lower cut-off level.

Since several studies in the literature present 0.1 mIU/mL as the best LH basal cut-off value [21, 30, 31, 43] we verified sensitivity and specificity for this level from our results, which were 93.53% and 61.54%, respectively. These results are consistent with those presented by Lee et al. [30], who found 88.4% sensitivity and 56.4% specificity. Conversely, Neely et al. and Houck et al. [21, 43] showed higher sensitivity and specificity (94% and 88%, 93% and 100%, respectively). Surprisingly, Pasternak et al. [31] found an inverted relationship between sensitivity (64%) and specificity (94%). On the other hand, Heo et al. found a cut-off value for basal LH of 0.245 mIU/L, which surprisingly had a 48% of specificity and a 88% of sensitivity [47].

So, according to the literature [43], our data confirms that FSH basal levels do not add any additional benefit in distinguishing between CPP, EP and PIT, although mean basal FSH levels were higher in CPP and EP compared with PIT group (*p* < 0.0001).

After analysis of the ROC basal LH/FSH ratio, we chose a value of 0.1 as cut-off to identify an HPG axis activation, with a sensitivity of 85.90% and a specificity of 78.14%. This cut-off is similar to that proposed by Binay et al. [44] of 0.08 (sensitivity – Se- 71.4%, specificity – Sp- 75.6%), but is very different from that presented by Pasternak et al. [31] of 0.05 (Se 71.0% and Sp 86.8%), and Lee et al. [30], who proposed a basal LH/FSH ratio of 0.04 (Se 54.4% and Sp 93.7%).

We decided to evaluate the predictability of combining basal LH levels ≥0.14 mIU/mL and LH/FSH ratio ≥0.1: our hypothesis was to measure LH/FSH ratio only in patients with an LH basal level above 0.14 mUI/mL, in order to increase the specificity of this diagnostic path. With this approach resulting sensitivity and specificity were 74.0% and 93.6%, respectively. Similarly, Chotipakornkul et al. have identified a higher cut-off value of basal LH of 0.2 mIU/L which combined with basal LH/FSH ratio (cutoff: 0.1) could easily and cost-effectively diagnose CPP in a girl in breast Tanner stage II, with 71.4% and 100% of sensitivity and specificity, respectively [46].

On the other hand, the Indian study conducted by Tripathy et al. has evaluated the role of urinary gonadotroping (uLH, uFSH) for the diagnosis of various pubertal disorders and in the monitoring of therapy in patients with CPP, finding that urinary gonadotropins strongly correlat with serum gonadotropins [49]. Specifically, The uLH level of ≥0.76 IU/L had 100% sensitivity and specificity to differentiate CPP from peripheral precocious puberty, whereas uLH level of ≥1.07 IU/L had 100% sensitivity and specificity for differentiating CPP from PT [49].

Tipically, an accelerated linear growth, increased uterine and ovarian size, advanced BA, and high spontaneous LH concentration were commonly observed in subjects with CPP [50].

On one side, pelvic ultrasouds has some well-known advantages, including being noninvasive, inexpensive, readily available, radiation-free, and reproducible, and is a very useful diagnostic tool for evaluating the paediatric and adolescent female pelvis [50]. It provides detailed information about the size of the uterus and ovaries, fundo-cervical ratio, endometrial thickness, and size and distribution of ovarian follicles [51].

But the role of obstetric ultrasound in predicting CPP is confusing: in 1993 Haber et al. [26] published reference values for uterine and ovarian size in girls between 1 day and fourteen years of age and concluded that an increase in size of the uterus is one of the very first signs of puberty in girls, since, with the exception of the first three months of life, this value is relatively stable until the beginning of pubertal development. For this reason, the same group proposed a cut-off value of uterine length of 36 mm (Se 90% and Sp 100%) and an ovarian volume cut-off of 1.2 ml (Se 82% and Sp 95%) [28]. In 2002 Herter et al. [24] published different best cut-off points in discriminating pre-pubertal girls from girls with CPP: uterine length 40 mm (Se 86% and Sp 100%), uterine volume 3 ml (Se 100% and Sp 93%) and ovarian volume 1 ml (Se 100% and Sp 100%). Some authors have found maximum values of uterine length ranging from 33 mm to 35 mm [52, 53], whereas others have found values greater than 40 mm [28, 54, 55]. Badouraki et al. [27], concluded that uterine length was the best parameter in distinguishing between CPP and PIT with a cut-off of 38.3 mm for girls aged 6 to 8 years (Se 82.4% and Sp 90.9%). For ovarian volume, the cut-off was found to be 3.35 ml for the same lippge group (Se 100% and Sp 89.5%). In 2006 De Vries found that uterine volume (>2 ml, Se 68.8%, Sp89.4%), uterine transverse diameter (>15 mm, Se 67.9%, Sp 100%), fundus diameter (>8 mm, Se 82.5%, Sp 76.4%) and ovarian circumference (>4.5 ml, Se 67.6%, Sp 85.7%) were significantly different between CPP and PIT [25]. In 2011 the same author [23] stated that pelvic ultrasound is not always sufficiently reliable to differentiate CPP from PIT because there is a significant overlap between normal pre-pubertal and early pubertal values, but could be used to improve diagnostic accuracy since the presence of uterine length greater than 40 mm, a transverse diameter greater than 15 mm and uterine volume >2 ml make the diagnosis of CPP very likely in girls with premature breast development [23]. In a recent consensus statement [18] it was reported that ultrasound could be helpful as an adjunct to GnRH stimulation in differentiating CPP from PIT and the authors reported cut-off levels for uterine length ranging from 34 to 40 mm, and for ovarian volume ranging from 1 to 3 ml. This wide variation in cut-off limits is probably due to different sample populations, different sample sizes, different statistical analysis and different skills in ultrasound assessment. Analysing our ROC for uterine length we found 35 mm to be the best cut-off, which had low sensitivity and specificity (83.45% and 42.86%, respectively), while ROCs for the ovarian volumes had even lower sensitivity and specificity values, so we decided to choose the cut-off derived from literature [39]. A test comprising both positive LH basal levels and pelvic ultrasound parameters gave a sensitivity of 82.1% and a specificity of 83.1%, which are both low.

These disappointing results in differentiating CPP and EP from PIT may be due to procedural bias. In fact, ultrasonography is strictly dependent on the operator and should always be performed by the same skilled specialist. The retrospective design of our study excluded this possibility.

Uterine length may not be the most reliable parameter for evaluating pubertal change in girls. Further studies are necessary to better determine the role of ultrasonography in the preliminary evaluation of girls with early signs of puberty since its role cannot be defined by our data.

On the other hand, advanced bone age is the most effective predictor of the result of luteinizing hormone-releasing hormone stimulation test [53]. A significantly higher BA-CA and faster growth were observed in subjects with positive results on the initial test when compared with subjects with negative test results in the follow-up test [53].

In conclusion, we believe that LH basal levels ≥0.14 mIU/mL could be very useful for avoiding unnecessary GnRH stimulating testing: due to the high sensitivity of this cut-off we had a false negative rate of only 9.4%, but a high percentage of false positives (21.9%).

Taking in consideration the incresead incidence of newly diagnosed CPP and the faster rate of pubertal progression in patients with a previous diagnosis not only during the lockdown due to Covid-19 pandemy but also in the following years, it could be useful to simplify the diagnostic means of this frequent condition [56, 57].

In our daily practice, in cases of suspected CPP we always perform a GnRH test but this is very expensive. A good screening test would allow us to avoid unnecessary specific diagnostic tests. A basal LH < 0.14 mIU/mL, obtained by a third-generation assay, excludes CPP thus avoiding the need for a GnRH stimulation test. In our population we found 31% of girls with suspected CPP and basal LH < 0.14 mIU/mL, among these 9.4% were false negatives and this implies that they will undergo a GnRH stimulation test later, during the follow up, allowing us to save 21.6% of useless GnRH tests.

A basal LH measurement can be obtained by the primary care physician to help to guide decisions about referring patients for additional subspecialty evaluations. When basal LH levels are above the cut-off limit and clinical findings are consistent with puberty, the diagnosis of CPP is very likely, conversely when basal levels are undetectable and the clinical picture is reassuring (minimal changes and lack of progression, pre-pubertal growth velocity, normal skeletal maturity), CPP can be ruled out. However, a close clinical and sonographycal follow-up is essential to intercept early those patients who have false negative basal LH test results and who may require a GnRH stimulation test later on.

Using this approach, the stimulation test should be indicated to confirm diagnosis in cases with high basal LH levels or to better understand a situation in which clinical indicators disagree with basal gonadotropin levels.
